# Ultrasound-Assisted Synthesis of SnS_2_ Quantum Dots Using Acetone as Solvent

**DOI:** 10.3390/ma18010082

**Published:** 2024-12-28

**Authors:** Grzegorz Matyszczak, Krzysztof Krawczyk, Albert Yedzikhanau, Cezariusz Jastrzębski, Piotr Dłużewski, Aleksandra Fidler, Tomasz Płociński, Krystyna Lawniczak-Jablonska, Anna Wolska, Aleksandra Drzewiecka-Antosik

**Affiliations:** 1 Faculty of Chemistry, Warsaw University of Technology, 00-664 Warsaw, Poland; 2 Faculty of Physics, Warsaw University of Technology, 00-662 Warsaw, Poland; 3 Institute of Physics, Polish Academy of Sciences, 02-668 Warsaw, Poland; 4 Faculty of Materials Science and Engineering, Warsaw University of Technology, 02-507 Warsaw, Poland

**Keywords:** sonochemistry, quantum dots, tin(IV) sulfide, nanomaterials, SnS_2_

## Abstract

A sonochemical synthesis of SnS_2_ quantum dots using acetone as a solvent is investigated. Two different tin sources (SnCl_2_∙2H_2_O or SnCl_4_∙5H_2_O) as well as two different sulfur sources (thioacetamide or Na_2_S_2_O_3_) were applied. The sonication time was also varied between 60 and 120 min. Resulting products of syntheses were characterized with the following techniques: powder X-ray diffraction, electron microscopy (SEM and HR-TEM), Raman and FT-IR spectroscopies, the Tauc method, and X-ray photoelectron spectroscopy. Obtained SnS_2_ nanostructures were in the form of quantum dots in the case of synthesis lasting 60 min (size of crystallites in the range of 3.5–7 nm) and in the form of elongated nanorods of length ca. 25–30 nm and width of 5–6 nm in the case of synthesis lasting 120 min. XPS analyses revealed that the surface of the obtained products contained a significant amount of tin at the second oxidation state (i.e., SnS). The quantum dots produced in the synthesis lasting 60 min showed a value of energy bandgap of 2.7 eV indicating potential applications in photocatalysis.

## 1. Introduction

Tin chalcogenides are a well-known group of inorganic chemical compounds. Their specific structural properties allow for the formation of very interesting, possible new mixed-valence tin chalcogenides [1]. For example, tin together with sulfur forms two basic tin sulfides (SnS and SnS_2_); however, they may be ‘mixed’ to form two other known tin sulfides (Sn_2_S_3_ and Sn_3_S_4_) [1,2,3]. These compounds are composed of SnS and SnS_2_ “units” mixed in certain proportions (e.g., 1:1 and 2:1 for Sn_2_S_3_ and Sn_3_S_4_, respectively) [2,3,4]. This fact contributes to the rich and interesting chemistry of tin chalcogenides [5].

Tin chalcogenides, due to their wide properties, are interesting also from the perspective of materials science. Tin(II) sulfide is a semiconductor with an optical energy bandgap lying typically in the range of 1.1–1.5 eV [6]. It is also characterized by p-type conductivity and a relatively large optical absorption coefficient. Thus SnS is an attractive material for utilization in fields such as photonics, photovoltaics, and optoelectronics [7,8]. The other two tin sulfides, Sn_2_S_3_ and SnS_2_, have a conductivity of n-type which is caused by sulfur vacancy related to the Sn(IV) oxidation state [6].

Wide applications of tin sulfides often require the preparation of these compounds in the form of nanostructures. Among the methods that have been used to prepare tin(II) and tin(IV) sulfide nanostructures, one can mention solvothermal (including hydrothermal) routes, the hot injection method, polyol methods, and precipitation from aqueous solutions [9,10,11,12,13,14,15,16]. However, these methods typically involve the usage of toxic high-boiling solvents and/or toxic reagents. A promising alternative for these methods is the sonochemical synthesis which meets the criteria of the so-called “Green Chemistry” [17]. The sonochemical synthesis was successfully used for the preparation of inorganic nanostructures of simple (e.g., ZnS, CdS) and complex (e.g., Cu_3_BiS_3_, CuInS_2_, Cu_2_ZnSnS_4_) inorganic compounds [18,19,20,21,22]. By the manipulation of the parameters of the sonochemical synthesis, one can achieve many morphologies and sizes of products; however, the control of the product may be additionally enhanced by merging sonochemical and electrochemical syntheses [23,24,25,26].

Tin sulfides were prepared via ultrasound-assisted synthesis many times. Nanoparticles of SnS were synthesized using direct sonication and reagents such as SnCl_2_, Na_2_S_2_O_3_ or Na_2_S, and CH_3_COONH_4_ in an unknown solvent [27,28,29]. Indirect sonication was also used in the sonochemical synthesis of SnS resulting in nanoparticles of 7 nm in the mean size, however, also in unknown solvent [30]. Using a mixture of ethanol and ionic liquid (1-buthyl-3methylimidazole tetrafluoroborane), and thioacetamide as a source of sulfur allowed for the synthesis of SnS with particles of sizes in the range from 50 to 700 nm [31]. On the other hand, starting from the same reagents but using ethylene glycol mixed with an amine (ethanolamine, diethanolamine or triethanolamine), it was possible to achieve SnS nanoparticles with sizes in the range of 4 to 15 nm [32]. There are fewer reports on the sonochemical synthesis of SnS_2_ in comparison with SnS. However, SnS_2_ nanoparticles of sizes in the range of 24–30 nm were prepared in aqueous solutions of SnCl_4_ and thioacetamide with the optional addition of concentrated hydrochloric acid [33,34,35]. Finally, quantum dots of both tin sulfides, with sizes varying from 1.5 to 10 nm for SnS_2_ and from 3 to 8 nm for SnS, were sonochemically obtained using the most technologically desirable solvent, which is water [36]. Despite the described approaches for the sonochemical synthesis of SnS and SnS_2_ (preferably in the form of quantum dots), the influence of the utilized solvent on the product is still unclear and more studies (using other solvents) need to be conducted.

This study presents the investigation of the possible application of acetone as a solvent in the sonochemical synthesis of SnS and SnS_2_ quantum dots. The following starting reagents were used: SnCl_2_ or SnCl_4_ (as a source of tin) and thioacetamide or sodium thiosulfate (as a source of sulfur). The resulting products of syntheses were isolated, purified, and subjected to investigations with the following techniques: powder X-ray diffraction, Raman and FT-IR spectroscopies, UV-Vis spectrophotometry (estimation of energy bandgap with the Tauc method), electron microscopy (SEM and HR-TEM), and X-ray photoelectron spectroscopy (XPS).

## 2. Materials and Methods

### 2.1. Materials and Reagents

All chemicals used in this study were pure for analysis (producer: POCH–Polskie Odczynniki Chemiczne, Poland). For sonochemical syntheses, SnCl_2_∙2H_2_O, SnCl_4_∙5H_2_O, Na_2_S_2_O_3_, and thioacetamide (TAA) were used as reagents. Acetone was used as a solvent in the syntheses and ethanol was used for the purification of prepared suspensions.

### 2.2. Sonochemical Syntheses

The sonochemical syntheses were conducted in conical flasks of 50 mL volume in an ultrasonic cleaner (PS 10A) generating an ultrasound of 40 kHz frequency with nominal power of ultrasounds 60 W. The acoustic power determined by the calorimetric method was 27.9 W/L.

20 mL of acetone was measured with a graduated pipette and placed in a flask. Weighed reagents (see Table 1 for detailed information on amounts) were placed in the solvent in the conical flask and the mixture then was stirred magnetically for 20 min. Next, the flasks were closed with glass stoppers and placed in the ultrasonic cleaner so the level of liquid in the cleaner was the same as the level of liquid in the flasks. The duration of sonication was 60 or 120 min.

Immediately after the reaction, the conical flasks were opened and kept under the laboratory hood for ca. 1 h to remove the potentially toxic gases produced during the reaction. After that, the produced powders were purified by subsequent centrifugations according to the following procedure: First, the reaction mixture was centrifuged and the supernatant was removed; next, the sediment was suspended in fresh ethanol (10 mL) in the ultrasonic cleaner for 10 min; when the obtained suspension has been centrifuged, the supernatant was removed and the sediment was suspended in a fresh portion of ethanol (10 mL) in the ultrasonic cleaner for 10 min (this point was additionally repeated 2 times). Such a procedure resulted in a suspension in ethanol of the synthesized powders.

### 2.3. UV-Vis Spectrophotometry

The UV-Vis spectra of diluted transparent suspensions in ethanol of synthesized powders were recorded within the wavelength range of 380–1000 nm using a spectrophotometer Model UV1600 (AOE Instruments, Shanghai, China). The collected spectra were subsequently used to perform analyses based on the Tauc method.

### 2.4. SEM Investigations

SEM observations were carried out on the Field Emission Scanning Electron Microscope (FE-SEM) made by Hitachi High Technologies company (Krefeld, Germany), model SU8000. The images were taken with an upper detector which is a semi-in-lens type detector, and provides the best resolution. The magnification range of 20,000× up to 100,000× at 5 keV and short working distance was used to determine the fine structure of particles.

### 2.5. Raman Spectroscopy

Raman measurements were conducted using an Aramis spectrometer of Horiba Jobin Ivon in backscattering geometry. The 633 nm line of the He-Ne–ion laser was used as the excitation. Raman spectra were collected using a 2400 L/mm diffraction grating and thermoelectric-cooled Synapse CCD at room temperature and under normal conditions. The spectral resolution of the measured spectra in this configuration was about 1 cm^−1^. A low-power laser beam was used to avoid thermal effects.

### 2.6. FTIR Spectroscopy

The Fourier-transform infrared spectroscopy measurements were conducted using NICOLET 6700 FT-IR spectrometer (Thermo Fisher Scientific, Waltham, MA, USA). Synthesized, dry powders were grounded with KBr and formed as pellets.

### 2.7. HR-TEM Investigations

One drop of the powder suspended in ethanol was deposited on a standard TEM copper grid coated with an amorphous carbon film. The grid was dried afterward and then used for the TEM investigation.

Investigations were conducted with the use of FEI Titan Cubed 80–300 TEM operating at 300 kV. The overview images were registered in bright-field TEM mode for magnifications ranging from 27,000× to 89,000×. For the purpose of high-resolution imaging, magnifications from 380,000× to 420,000× range were used. In both cases, the images were obtained with the Gatan BM-Ultrascan CCD camera.

### 2.8. XPS Investigations

The suspensions of samples were dripped on Cu conductive tape and left to dry in a laboratory fume cupboard. The procedure was repeated until a visible sample layer was obtained then samples were introduced to the spectrometer. X-ray photoelectron spectra (XPS) were recorded by a Prevac setup equipped with a high-intensity monochromatic X-ray Al Kα (1486.69 eV) source Scienta MX 650 (set at 300 W), Scienta R4000 hemispherical analyzer, and charge neutralization. The narrow scans were acquired with pass energy 200 eV and the step 0.2 eV. The full width at half maximum (FWHM) for the Au 4f 7/2 line measured at the same experimental condition was equal to 0.6 eV. The energy scale was calibrated setting the C 1 s line at the position 285.0 eV. Spectra were analyzed using the commercial CASA XPS software package (Casa Software Ltd., version 2.3.17) with Tougaard background and a GL (30) line shape (70 % Gaussian, 30 % Lorentzian). The wide spectra were registered with pass energy 500 eV and the step 0.5 eV.

## 3. Results and Discussion

According to data presented in Table 1, only two experimental setups led to the formation of precipitates that were possible to separate by the centrifugation. Such yellow precipitates had a color characteristic for tin(IV) sulfide (SnS_2_) which cannot be matched with any other known tin sulfide (SnS—dark brown, Sn_2_S_3_—brown). Yellow precipitates were obtained using SnCl_2_∙2H_2_O as the tin source and thioacetamide as the sulfur source, with no relation to the sonication time. Using the same sulfur source but with SnCl_4_∙5H_2_O as the tin source, the reaction mixture turned to yellow color after the sonication but no precipitate was possible to separate by the centrifugation. The opacification was greater in the case of longer sonication time. The resulting yellow color without separable precipitate indicates the formation of ultrasmall nanoparticles of SnS_2_ with probably a greater yield in the 120-min-long synthesis. On the other hand, in the case of the usage of Na_2_S_2_O_3_ as the sulfur source, the reaction mixtures remained unchanged even after 120 min of sonication which indicates no reaction, at least toward the formation of tin sulfides.

**Figure 1:**
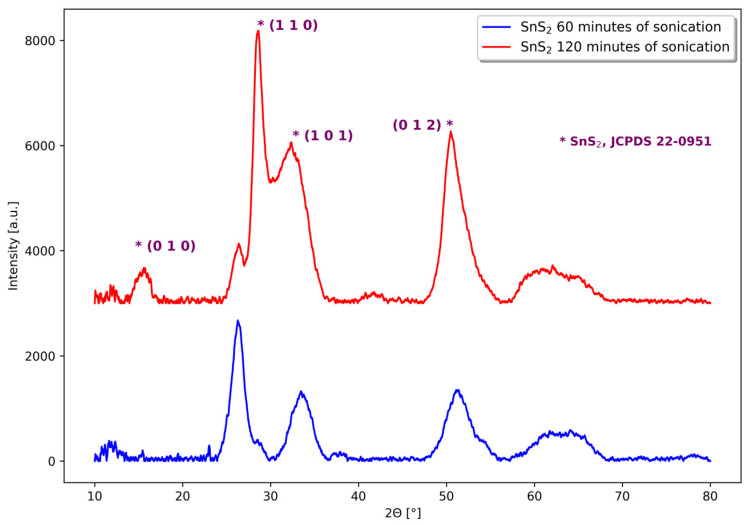
X-ray powder diffractograms of yellow precipitates obtained in the sonochemical syntheses performed in acetone using SnCl_2_∙2H_2_O and thioacetamide as reagents and with sonication time 60 min (bottom curve) and 120 min (upper curve).

The results of the X-ray powder diffraction investigations conducted on the two yellow precipitates suggest the presence of tin(IV) sulfide in the nanocrystalline form. This is indicated by the broadening of the reflexes in the powder diffractogram, as well as by the absence of the large and wide peak in the range of angle from 10 to 15° (see Figure 1).

**Figure 2:**
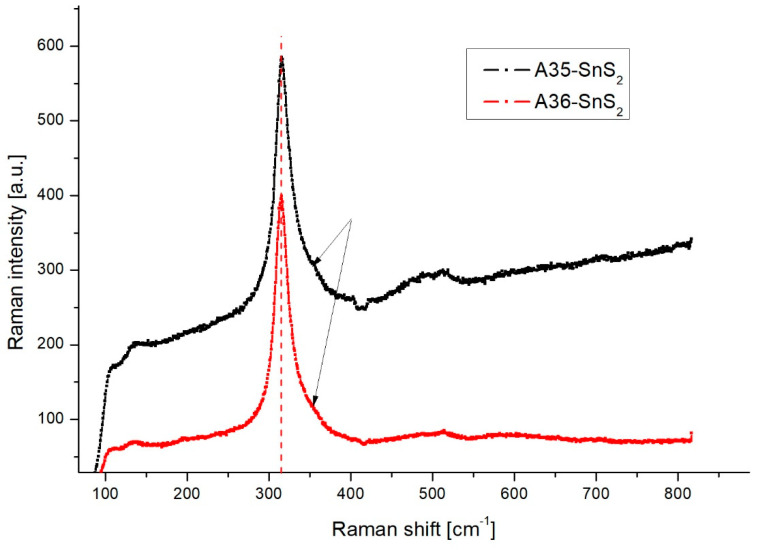
Typical Raman spectra of samples A35 and A36. A vertical dashed line was placed for 315 cm^−1^. Arrows indicate asymmetry of the A_1g_ peak.

Raman studies confirmed the formation of SnS_2_ nanograins in the sonochemical synthesis process. There were no precipitations of another phase. The Eg and A1g symmetry peaks indicate a trigonal crystal structure of SnS_2_ with a 2H polytype. This is evidenced mainly by a clear peak of A_1g_ for about 315 cm^−1^ (Figure 2) [37,38]. The intensity of the E_g_ peak at about 205 cm^−1^ is very low and hardly observed.

A slight shift of this peak of about 0.5 cm^−1^ toward higher frequencies in the case of the A35 sample compared to the A36 sample was observed. This may be attributed to small amounts of polytypes different from 2H in the A35 sample [39]. Some asymmetry of the peak was observed for both types of samples. Such asymmetry may be caused by phonon-free carriers interaction or is due to phonon constraints in nanostructures [40]. In the case of SnS_2_, this broadening may result also from the disorder activation of forbidden phonons with A_2u_ symmetry [41].

The asymmetry of the A_g_ peak is greater in the case of A35 samples. At the same time, a much higher luminescence background is observed for these samples. Hence, it can be concluded that the broadening of the A_g_ peak results from a higher concentration of defects and activation of phonons normally forbidden by the selection rules.

**Figure 3:**
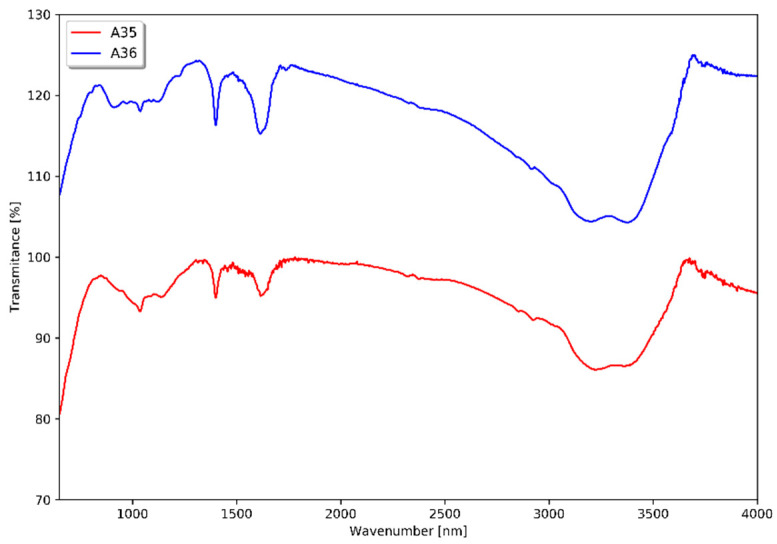
FT-IR spectra of samples A35 and A36.

Additional FT-IR spectroscopy investigations were conducted to confirm the presence of tin(IV) sulfide (Figure 3). In both yellow precipitates, Sn-S bonds are presented (region of 1000–1300 cm^−1^). The FT-IR spectra show the occurrence of trace amounts of solvent used in purification (C_2_H_5_OH) as indicated by peaks corresponding to C-C and C-H bonds, and -OH groups (1400, 1600, and 3000–3500 cm^−1^). The bands related to Sn-S bonds are similar in both samples of SnS_2_ which is in line with the XRD and Raman results.

**Figure 4:**
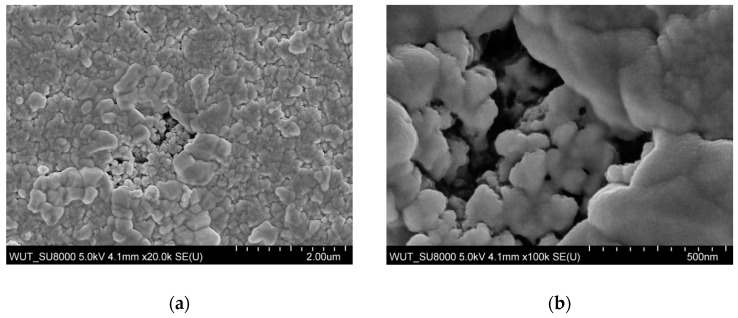

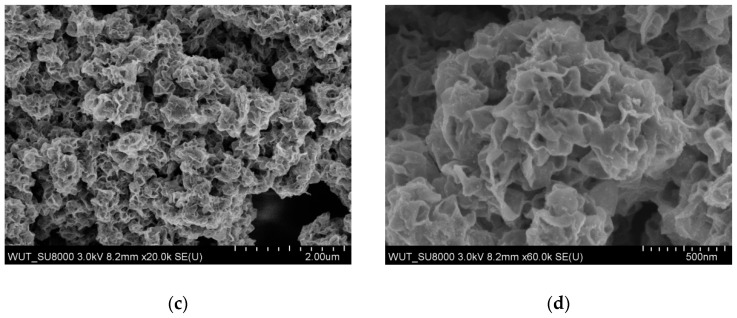
SEM images of dried products of syntheses with duration: (**a**,**b**) 60 min; (**c**,**d**) 120 min.

The morphology of the dried yellow precipitates was investigated by the scanning electron miscroscopy (SEM) observations. The dried product of reaction that durated 60 min formed submicron agglomerates (Figure 4a) made of nanometric particles (Figure 4b). On the other hand, the dried product of a 120-min-long reaction formed submicron particles with interesting, flower-like morphology (Figure 4c). Observations of higher magnitude revealed that the flower-like particles are made of elongated and narrow nanostructures (Figure 4d). The EDX elemental analyses confirmed the presence of desired elements (Sn and S) in the obtained powders, however, with deviations from the ideal atomic ratio of Sn to S which in bulk SnS_2_ is 1:2. For the powder obtained in the 60-min-long synthesis, the Sn:S ratio is 1.75:1, while for the powder obtained in the 120-min-long synthesis, the Sn:S ratio is 0.73:1. The difference from the ideal ratio may be likely caused by the formation of nanoparticles in the syntheses; however, it may be seen that with longer sonication time, the Sn:S ratio changed its value toward the ideal one.

**Figure 5:**
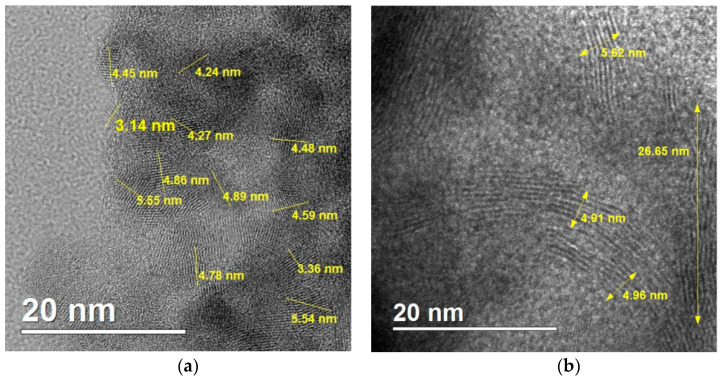
HR-TEM images of the products of syntheses with duration: (**a**) 60 min; (**b**) 120 min.

Observations made by the HR-TEM technique proved the presence of nanocrystallites in the prepared samples of SnS_2_ (Figure 5). In the product of synthesis lasting 60 min, nanocrystallites with sizes in the range from 3.5 to 7.5 nm were confirmed (Figure 5a). Elongated and narrow nanostructures were present in the sample obtained by 120-min-long sonication (Figure 5b).

**Figure 6:**
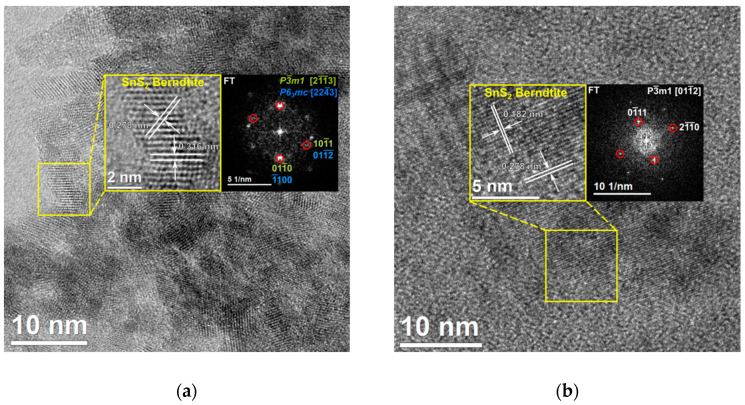
HR-TEM images, together with Fourier transforms, of dried products of syntheses with duration: (**a**) 60 min; (**b**) 120 min. (Red circles indicate spots corresponding to the structure of the analyzed nanocrystal; other spots come from neighboring ones).

Further observations with HR-TEM technique and analyses based on their Fourier transforms confirm that nanocrystallites have structure typical for SnS_2_ (Berndtite structure, see Figure 6).

**Figure 7:**
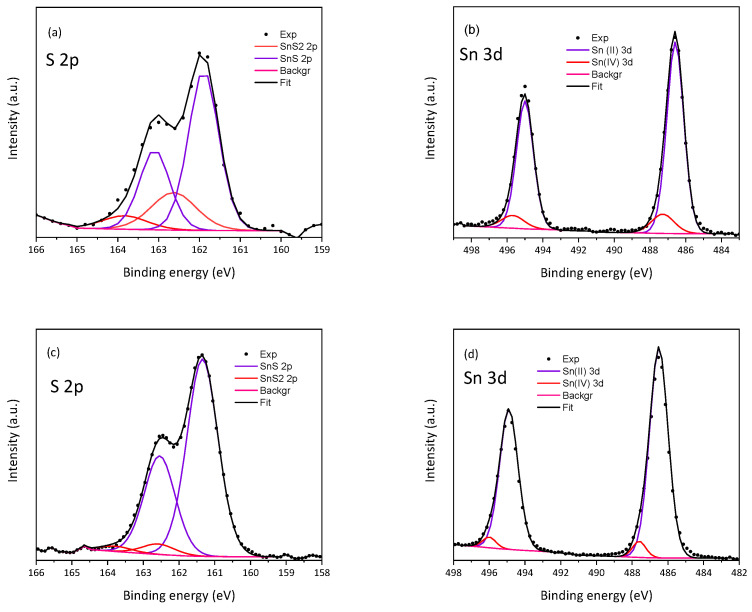
XPS spectra of S 2p (**a**,**c**) and Sn 3d (**b**,**d**) of sonochemically synthesized SnS_2_ quantum dots: (**a**,**b**) 60-min-long synthesis; (**c**,**d**) 120-min-long synthesis.

The results of the X-ray photoelectron spectroscopy investigations are presented in Table 2 and Table 3, and in Figure 7. Like the results of the EDX elemental analysis, they indicate that the Sn:S ratio evolves toward an ideal ratio of value 1:2 with longer synthesis times. However, it turns out that on the surface of both samples, the main component is not SnS_2_, but SnS. Additionally, the fraction of SnS_2_ on the surface of the product decreases with increasing sonication time. Quite high values of FWHM and a slightly changed binding energy of the fitting curves indicated a large disorder in the chemical structure of the formed nanoparticles, therefore, we cannot exclude the possibility of the formation of other compounds such as, e.g., Sn_2_S_3_ or Sn_3_S_4_.

**Figure 8:**
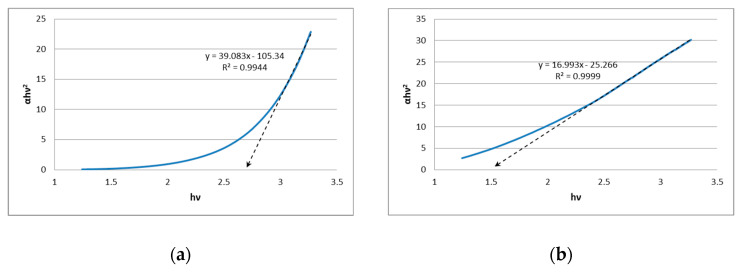
The Tauc plots of sonochemically synthesized SnS_2_ quantum dots: (**a**) 60-min-long synthesis; (**b**) 120-min-long synthesis.

The analyses performed basing on the Tauc approximation revealed that the product of synthesis lasting 60 min has a value of energy bandgap for direct transition of 2.7 eV (see Figure 8a for relevant Tauc plot). Such a value is slightly greater than the value corresponding to the bulk SnS_2_ which is ca. 2.4 eV [42]. The increase in the energy bandgap is likely caused by the quantum confinement effect. On the other hand, the product of synthesis lasting 120 min is characterized by the energy bandgap of value 1.5 eV for direct transition (see Figure 8b) exhibiting a significant difference in comparison with the bulk SnS_2_. Such a result may suggest the presence of other phases (SnS, Sn_2_S_3_, Sn_3_S_4_) in the prepared powder of SnS_2_. According to the XPS results, the chemical state on the surface of QDs prepared in the 120-min-long synthesis is mainly Sn(II) suggesting the passivation of SnS_2_ with a layer of SnS. This passivation likely causes the decrease in the measured value of the optical energy bandgap in comparison with the bulk value. Similar behavior may be observed for the passivation of SnS particles with SnO, which, in such cases, leads to the increase in the observed optical energy bandgap. It should be also noted that the nanoparticles in the product of a 120-min-long synthesis are significantly larger thus the quantum confinement is reduced (in comparison to a product of synthesis lasting 60 min). The values of optical energy bandgaps suggest possible applications of prepared quantum dots in the field of photocatalysis (product of synthesis lasting 60 min; relatively high value of 2.7 eV) and photovoltaics (product of synthesis lasting 120 min; value 1.5 eV lying in the so-called Schockley–Queisser limit [43]).

## 4. Conclusions

We present a procedure for the sonochemical synthesis of SnS_2_ quantum dots using, for the first time, acetone as a solvent. By doing so, we avoid the usage of high-boiling toxic organic solvents which complies with the “Green chemistry” approach. Different tin and sulfur sources were investigated and it turned out that SnS_2_ nanostructures may be obtained using SnCl_2_∙2H_2_O and thioacetamide. Obtained SnS_2_ nanostructures were in the form of quantum dots in the case of synthesis lasting 60 min (size of crystallites in the range of 3.5–7 nm) and in the form of elongated nanorods of length ca. 25–30 nm and width of 5–6 nm in the case of synthesis lasting 120 min. XPS analyses revealed that on the surface, the obtained products contained a significant amount of tin in the second oxidation state (i.e., SnS). The estimated values of energy bandgaps suggest potential applications of synthesized quantum dots in the field of photocatalysis (product of 60-min-long synthesis—2.7 eV) and photovoltaics (product of 120-min-long synthesis—1.5 eV). Investigated tin-based nanomaterials may be used as alternatives for Cd-containing quantum dots.

## Figures and Tables

**Table 1:** Summary of experimental conditions of conducted sonochemical syntheses.

No. (and Sample Name If Available)	Tin Source	Amount of Tin Source (mg)	Sulfur Source	Amount of Sulfur Source (mg)	Sonication Time (min)	Result of Synthesis
1	SnCl_4_∙5H_2_O	702	Thioacetamide	376	60	Clear, yellow solution (no precipitate after centrifugation)
2	SnCl_4_∙5H_2_O	702	Thioacetamide	376	120	Yellow suspension (no precipitate after centrifugation)
3 (A35)	SnCl_2_∙2H_2_O	452	Thioacetamide	376	60	Yellow precipitate
4 (A36)	SnCl_2_∙2H_2_O	452	Thioacetamide	376	120	Yellow precipitate
5	SnCl_4_∙5H_2_O	702	Na_2_S_2_O_3_	1242	60	Reaction mixture unchanged
6	SnCl_4_∙5H_2_O	702	Na_2_S_2_O_3_	1242	120	Reaction mixture unchanged
7	SnCl_2_∙2H_2_O	452	Na_2_S_2_O_3_	1242	60	Reaction mixture unchanged
8	SnCl_2_∙2H_2_O	452	Na_2_S_2_O_3_	1242	120	Reaction mixture unchanged

**Table 2:** Results of the XPS elemental composition (at %) of tested samples from high- and low- (survey) resolution spectra.

Sonication Time (min)	Spectrum Resolution	Sn	S	C	O	S:Sn
60	High	14.7	12.5	17.5	55.3	0.85
60	Low	14.3	15.4	20.0	50.3	1.1
120	High	7.2	9.5	70.2	13.1	1.3
120	Low	0.74	1.6	77.5	20.2	2.2

**Table 3:** The binding energy (eV); percentage of given fraction, and full width at half maximum (eV) (FWHM) for chemical components in analyzed samples. FWHM indicates the level of chemical order in formed compounds.

Sonication Time (min)	BE of S in SnS	FWHM	BE of S in SnS_2_	FWHM	BE of Sn(II) in SnS	FWHM	BE of Sn(IV) in SnS_2_	FWHM
60	161.9; 74.1%	0.9	162.6: 25.9%	1.3	486.6; 88.2%	1.1	487.3; 11.9%	1.5
120	161.3; 94.8%	1.0	162.6; 5.2%	1.1	486.5; 94.8%	1.2	487.6; 5.2%	0.9

## Data Availability

Dataset available on request from the authors.
